# Pathogenesis of Chronic Arthritis Due to Chikungunya Virus and Advances in Vaccine Development

**DOI:** 10.3390/v18040428

**Published:** 2026-04-01

**Authors:** Meng Ma, Leyi Li, Hao Sun, Xiaochao Zhang

**Affiliations:** 1 Yunnan Provincial Key Laboratory of Pharmacology for Natural Products, School of Pharmaceutical Sciences, Kunming Medical University, 1168 Yuhua Road, Kunming 650500, China; mameng0126@163.com (M.M.); lileyi1108@163.com (L.L.); 2 Medical School, Kunming University of Science and Technology, 727 Jingming South Road, Kunming 650500, China; 3 Institute of Medical Biology, Chinese Academy of Medical Sciences & Peking Union Medical College, 935 Jiaoling Road, Kunming 650118, China

**Keywords:** chikungunya, chronic arthritis, pathogenesis, vaccine, immunopathogenesis, epidemiology

## Abstract

Chikungunya virus (CHIKungunya Virus, CHIKV) is a mosquito-borne plus-stranded RNA virus. Adaptive mutations such as A226V in the E1 envelope protein of CHIKV significantly enhance the transmission efficiency of the virus in *Aedes albostriae*, leading to multiple rounds of epidemics around the world including the large-scale outbreak in Guangdong Province in 2025. After a viral infection, a significant proportion of patients will progress from acute arthralgia to chronic arthritis that persists. The pathogenesis of the disease involves the persistence of the virus in joint tissues, the persistent inflammatory response with IL-1β, IL-6 and IL-17 as the core mediated by macrophages, possible autoimmune cross-reactions, and individual genetic susceptibility. At present, there is no specific antiviral drug, but important progress has been made in vaccine development against the virus. Vaccines based on live attenuated virus (VLA1553) and virus-like particle (VLP) platforms have been approved for the market and provide a tool to prevent and control this important public health threat. This review synthesizes current knowledge on CHIKV-induced chronic arthritis pathogenesis and recent vaccine advances, providing a framework for understanding disease mechanisms and guiding future prevention strategies.

## 1. Structure and Epidemiology

This narrative review aims to systematically summarize the recent progress in the structure, epidemiology, replication, pathogenesis, and prevention of chikungunya virus. The critical nature of the innate immune response is emphasized for the virus, especially focusing on chronic arthritis and vaccine development.

The articles included in this review were selected through a comprehensive search of PubMed, Web of Science, and Google Scholar databases, using keywords such as “chikungunya”, “chronic arthritis”, “pathogenesis”, “vaccine”, and “interferon”. Only peer-reviewed articles published between 2008 and 2025 were considered, with priority given to recent and high-impact studies.

The genetic material of the chikungunya virus (CHIKV) is a single-stranded positive-sense RNA of approximately 11.8 kb in length. Its genomic structure closely mimics the mRNA of the host cell, featuring a 5′ methylated cap and a 3′ poly-A tail, enabling it to directly utilize the host ribosomes to initiate protein synthesis [1,2].

The CHIKV genomic RNA is translated to produce a non-structural polyprotein (nsP1–nsP4). During replication, a subgenomic RNA is generated, which serves as the mRNA for the structural polyprotein (C–E3–E2–6K–E1) [3]. These polyproteins are subsequently cleaved by viral and host proteases to yield mature functional proteins. The part located at the 5′ end, accounting for approximately two-thirds of the total length, encodes the non-structural proteins (nsP1–nsP4), which together constitute the RNA replication machinery of the virus [4]. The non-structural proteins mainly function during the early stage of viral replication, responsible for the replication of the viral genome and the processing of proteins [5]. The first one-third of the genome’s 3′ end encodes structural proteins, whose translation depends on a subgenomic RNA produced during the late stage of viral replication. The polyprotein translated from this RNA is then cleaved to produce capsid proteins (C) and envelope glycoproteins (E1, E2, etc.). The capsid protein is responsible for enclosing the RNA to form the nucleocapsid. In addition to the standard structural proteins, a transframe (TF) protein is produced as a result of ribosomal frameshifting within the 6K gene, which has been implicated in viral neuropathogenesis and assembly. This TF protein corresponds to a fusion product known as C-pE2-6K/TF-E1, generated by +1 ribosomal frameshifting during translation of the structural polyprotein [6]. Additionally, the envelope glycoprotein E2 is initially synthesized as part of a precursor called preE2 (or pE2), which is later cleaved by host furin-like proteases in the Golgi apparatus to yield mature E2 and E3 [7]. Both preE2 processing and TF-E1 production are critical for proper virion assembly and infectivity. The structural proteins constitute the physical structure of the virus and mediate the process by which the virus enters the host cell [8,9].

Among the non-structural proteins, nsP1 possesses methyltransferase and guanylate transferase activities. It can add a 5′ cap structure to the viral RNA, protecting the RNA from degradation and ensuring that it can be recognized by the host cell’s translation mechanism [10]. Furthermore, it also participates in anchoring the replication complex to the host cell membrane. nsP2 is a multifunctional protein that possesses both protease and helicase activities, and is responsible for cutting the viral polyprotein precursor to generate other non-structural proteins with functional properties [11]. At the same time, it can also suppress the host’s interferon response, helping the virus evade immune attacks. This makes nsP2 a potential target for neutralizing antibody research [12]. nsP3 plays a crucial role in the assembly and stability of the viral replication complex. It specifically recruits host proteins (such as G3BP1/2) through its C-terminal region and regulates RNA synthesis through interactions. This process not only disassembles the host’s antiviral stress granules but also inhibits the production of type I interferons, creating a favorable environment for viral replication [13,14,15,16]. nsP4 is an RNA-dependent RNA polymerase (RdRp), serving as the core catalytic enzyme for viral replication. It is responsible for synthesizing the viral genome RNA and subgenomic mRNAs, and thus is often selected as a key target for the design of antiviral drugs [17,18].

In terms of structural proteins, the E1 and E2 glycoproteins form spikes on the surface of the virus. These two proteins first form an heterodimer, and then assemble into a trimer [19]. The E2 protein is the main antigenic protein on the surface of the virus. During the infection process, E2 can directly recognize and bind to the surface receptors of the host cell. Subsequently, in the acidic environment of the endosome, E1 mediates the fusion of the viral envelope with the cell membrane, completing the invasion [20]. The E1 protein is a fusion protein. It undergoes conformational changes mainly in an acidic environment, mediating the fusion of the viral envelope and the cell membrane, allowing the viral genome to enter the cytoplasm [21]. E1 is often used in conjunction with E2 for the construction of virus-like particle vaccines to simulate the natural structure of viruses and enhance the immune response [22]. The C protein (capsid protein), located inside the virus, is mainly responsible for recognizing and packaging the viral RNA to form the nucleocapsid. It also connects the capsid to the viral envelope through interaction with the E2 protein, promoting the final assembly of the virus particle [23]. E3 proteins normally act as chaperones of E2, assisting in its proper folding and stabilizing its conformation [24]. The 6K protein, as an auxiliary structural protein, mainly participates in the budding process of the virus and the formation of the envelope, ensuring the correct positioning and function of E1 and E2 on the surface of the virus [25,26].

The global epidemic of CHIKV is the result of the interaction between its genome evolution and human activities. The virus was mainly reflected in four lineages: West African lineage (WA), East/Central/South African lineage (ECSA), Indian Ocean lineage (ILO), and Asian lineage (Asian). There were significant differences in geographical distribution, molecular characteristics, and epidemic patterns among these lineages [27]. CHIKV was first discovered in Tanzania in 1952 and is mainly confined to the forest ecosystems in western Africa, maintaining a “jungle cycle” pattern with non-human primates and forest-dwelling mosquitoes (such as *Aedes furcifer*) as the main hosts. It exhibits endemicity, has a relatively conservative genome, lacks key adaptive variations, and thus rarely causes large-scale urban epidemics [28]. In contrast, the ECSA lineage possesses remarkable urban adaptability [29]. In the early 21st century, the evolution of viruses witnessed a crucial turning point: around 2004, an emerging branch from the ECSA lineage—the Indian Ocean lineage (IOL)—emerged along the coast of Kenya [30,31]. The distinctive feature of IOL is that an amino acid substitution of E1-A226V occurs on the envelope protein E1 [32]. This mutation significantly enhanced the replication and transmission of the virus in Ae. albopictus by altering the dependence of the virus on cell membrane cholesterol [33]. Mutations such as L210Q and K252Q in E2 protein further consolidated its transmission advantage in *Aedes albopictus* [34]. This molecular adaptation has enabled IOL to cause a global epidemic since 2005, first causing large outbreaks on Indian Ocean islands such as La Reunion, then landing in India, causing the country’s worst outbreak in decades, and rapidly spreading to Southeast Asia [35,36]. Subsequently, there were large outbreaks of *Aedes albopictus* mosquitoes as vectors occurred in Italy and other European countries. The Asian lineage was first introduced to the Americas in 2013. From the island of St. Martin, the ECSA/IOL lineage was also introduced to Brazil and other places. Within a few years, the ECSA/IOL lineage had spread to more than 40 countries, causing more than one million infections [37]. At the same time, the main vector of CHIKV in Africa also changed from *Aedes aegypti* to *Aedes albopictus*, which accelerated the spread of the virus in Africa and completely changed the global epidemic pattern of CHIKV [38]. Since then, the virus has erupted in succession around the world, forming a complex epidemic situation involving simultaneous transmission, co-circulation, competition, and even substitution of multiple genetic lineages [39]. The introduction of the Asian lineage into Brazil remains a subject of academic debate. While initial genomic surveillance suggested a single introduction event from the Caribbean, alternative hypotheses propose multiple independent introductions from Pacific islands, highlighting the complexity of transcontinental viral dispersal.

**Figure 1:**
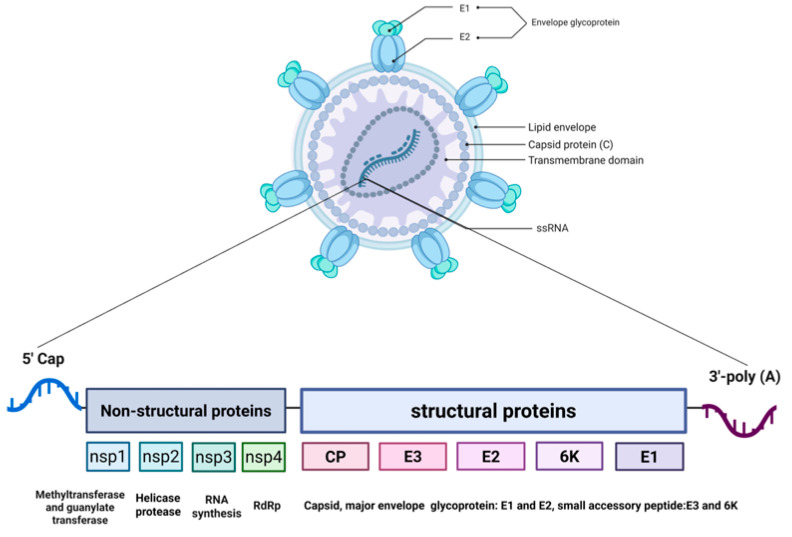
Schematic representation of the chikungunya virus (CHIKV) virion structure and genome organization. The upper panel illustrates the structure of the CHIKV virion, which consists of a single-stranded positive-sense RNA (ssRNA) genome encapsidated by capsid proteins (C), surrounded by a lipid envelope embedded with envelope glycoproteins E1 and E2. The lower panel shows the genomic organization of CHIKV. The 5′ capped RNA genome contains two main open reading frames (ORFs): the first encodes the non-structural polyprotein (nsP1–nsP4), which is processed into proteins responsible for RNA capping (nsP1), helicase/protease activity (nsP2), replication complex assembly (nsP3), and RNA-dependent RNA polymerase activity (nsP4); the second ORF encodes the structural polyprotein, which is cleaved into capsid protein (C), envelope glycoproteins (E3, E2, 6K, E1), and small accessory proteins. The 3′ end is polyadenylated. Abbreviations: CP, capsid protein; RdRp, RNA-dependent RNA polymerase. Created in BioRender. RUI, P. (2026) https://BioRender.com/ofcwc2g (accessed on 26 March 2026).

**Figure 2:**
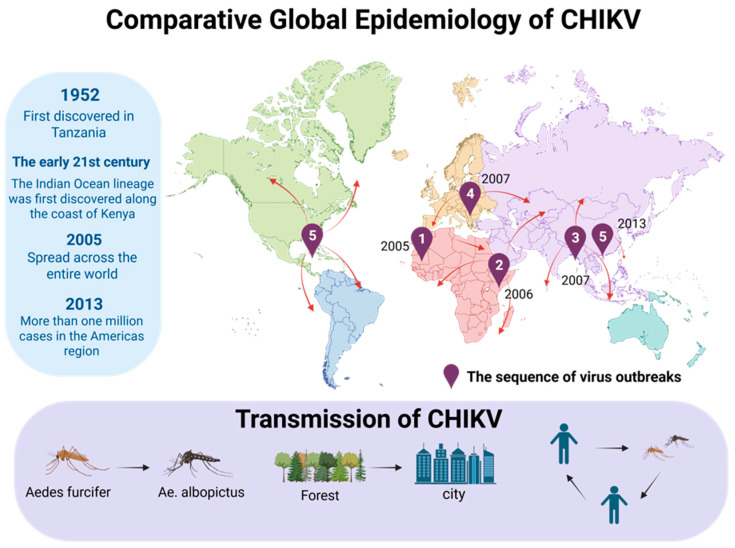
Global spread of the chikungunya virus (CHIKV) driven by the Indian Ocean lineage (IOL). The numbered markers (1–5) indicate the chronological sequence of major outbreaks caused by the IOL, which emerged in 2004–2005 and acquired enhanced transmission via *Aedes albopictus*. The inset illustrates the transmission cycle from forest to urban settings and the key mosquito vectors involved. Note that CHIKV was first isolated in Tanzania in 1952, but the modern pandemic is primarily driven by the IOL. The numbered markers (1–5) indicate the chronological sequence of major outbreaks caused by the IOL, with colors distinguishing different outbreak events and regions. Created in BioRender. RUI, P. (2026) https://BioRender.com/c7m4vi0 (accessed on 26 March 2026).

CHIKV is a mosquito-borne positive-sense single-stranded RNA virus. Its virion structure and genome organization are illustrated in Figure 1. The global transmission history and key vectors of CHIKV are summarized in Figure 2. In the 1980s, the CHIKV strain was isolated from the Yunnan region of China. In 1991, antibodies against CHIKV were detected in Hainan Province. In 2018, the first imported case of CHIKV was reported in the Chinese mainland [40]. The IOL strain carrying the E1-A226V mutation caused the first local outbreak of *Aedes albopictus* in Dongguan City, Guangdong Province, China in 2010, indicating that this lineage had established a stable transmission chain in the distribution area of *Aedes albopictus* [41]. In 2025, imported CHIKV cases were detected in Guangdong Province, which triggered the largest CHIKV outbreak in China thus far. This outbreak affected 21 cities, and more than 6000 cases were reported in Foshan alone in July [42,43]. As of 18 October 2025, a total of 16,738 confirmed cases had been reported in Guangdong Province, all of which were mild cases and no severe cases or deaths. The latest research confirmed that the pathogen of this outbreak was of the ECSA lineage, and confirmed that the sequence was similar to that of the S27b03 strain, carrying about 3879 C > T substitution, and that the isolates may have a common origin. This provided the conditions for the efficient transmission of the virus, and also reflected the ability of the ECSA lineage to use human movement to achieve fulminant transmission [44]. The epidemic of CHIKV has obvious seasonality, mainly occurring from July to November in China. Patients and inapparent infection cases are the main sources of infection, which are transmitted by the bites of *Aedes aegypti* and *Aedes albopictus*, forming a “human–mosquito–human” mode, and the population is generally susceptible [45]. Natural factors such as climate warming and increased rainfall will expand the distribution of Aedes mosquitoes. Social factors such as urbanization, insufficient monitoring capabilities, and increased international travel will all contribute to the spread and importation risks of the virus [46]. In summary, the current CHIKV has undergone key mutations and has adapted highly to the widely distributed *Aedes albopictus*. As a result, it has become the most threatening epidemic strain in terms of public health in Asia, Africa, and globally.

## 2. The Replication Process of CHIKV

**Figure 3:**
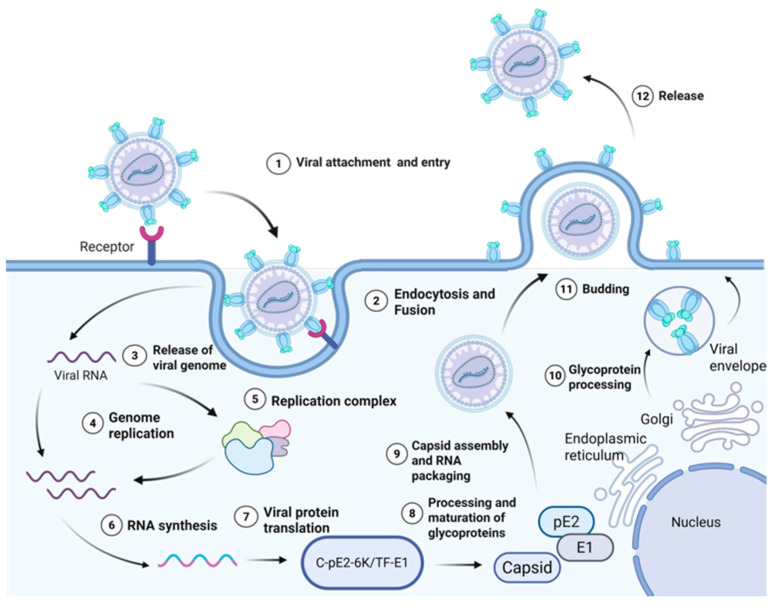
Replication and structural protein processing of the chikungunya virus (CHIKV). The viral genomic RNA is translated into non-structural (nsP1–nsP4) and structural polyproteins. The structural polyprotein (C–E3–E2–6K–E1) is cleaved by host and viral proteases to yield mature proteins. preE2 (pE2) serves as the precursor for E2 and E3 glycoproteins, while C-pE2-6K/TF-E1 is a frameshift-derived fusion protein generated via +1 ribosomal slippage in the 6K gene. Both preE2 processing and TF-E1 production are essential for virion assembly and infectivity. Created in BioRender. RUI, P. (2026) https://BioRender.com/jmplizh (accessed on 26 March 2026).

The replication cycle of CHIKV, including viral entry, genome replication, protein processing, and virion release, is illustrated in Figure 3. The life cycle of CHIKV involves a series of closely linked biochemical and cellular events, which is based on the efficient replication of the viral genome in the host cytoplasm. The infection depends on the recognition and binding of E2 glycoprotein on the surface of the viral envelope to specific receptor molecules on the cell membrane. After binding, the virus is mainly internalized into cells through the clathrin-dependent endocytic pathway [47]. As the endosomal compartments become acidic, the viral envelope fuses with the endosomal membrane, causing the internal nucleocapsid structure to be released into the cytoplasm. Subsequently, the positive strand RNA genome of the virus is released and is immediately read as a template by the host cell, translating to produce a non-structural polyprotein precursor nsP1234. The generation of this translation product marks the formal activation of the viral replication program [48].

The core link of viral replication is tightly regulated, and the key is the protease activity of nsP2 protein. nsP2 produces an early replicase complex consisting of a P123 polypeptide and nsP4 protein through an ordered proteolytic processing of the polyprotein precursor [49]. The replication process begins with the translation of the full-length non-structural polyprotein nsP1234 from the viral genomic RNA. This precursor is partially processed by the protease activity of nsP2 to generate nsP123 and mature nsP4. The accumulation of nsP123 together with nsP4 forms the early replication complex (RC), which is responsible for synthesizing the negative-strand RNA intermediate. Subsequent complete autoproteolytic cleavage of nsP123 into individual nsP1, nsP2, and nsP3 proteins triggers a conformational switch in the RC, redirecting its activity toward the production of genomic and subgenomic positive-strand RNAs [50]. This complex is capable of catalyzing the synthesis of complementary full-length negative-strand RNA using the initial input plus strand genome as a template [51]. At the same time, viral nonstructural proteins, especially nsP1, induce remodeling of the host cell inner membrane structure, usually forming vesicular membrane structures about 50–70 nm in diameter derived from endosomal or plasma membranes. These structures, known as “ replication spherules”, create a proprietary microenvironment for viral RNA synthesis [52].

nsP3 plays a key role in the assembly and function of the replication complex. It specifically recruits the host protein G3BP1 through the FGDF motif contained in its C terminus. This interaction not only localizes G3BP1 to the site of viral replication but also disrupts its normal function involved in the formation of stress granules [53].

As the nsP2 protease completely processes the polyprotein precursor into the mature nsP1, nsP2, nsP3, and nsP4 individual proteins, the composition and function of the replication complex undergo a transformation [54]. At this point, the mature replicase instead uses the newly synthesized minus-strand RNA as a template to produce two classes of plus-strand RNA products in large quantities: full-length genomic RNA and a shorter 26S subgenomic RNA. The full-length RNA will serve as the genome of the progeny virus. The 26S RNA encodes the structural proteins of the virus [55]. The translation products of structural proteins follow different processing and transport pathways. The capsid protein is synthesized in the cytoplasm, released by self-cleavage, and retained in the region. In contrast, the glycoprotein precursors enter the endoplasmic reticulum, undergo folding, glycosylation, and are cleaved by host proteases into mature E1, E2, and 6K proteins [56]. The mature E1–E2 heterodimer is eventually transported to the cell membrane for localization [57].

In the final stage of replication, capsid proteins in the cytoplasm specifically package the newly synthesized full-length genomic RNA and assemble into nucleocapsids. These nucleocapsids move to the inner side of the modified cell membrane and exit the host cell in a budding manner by interacting with the E1–E2 glycoprotein complex that is clustered there. The released viral particles acquire an envelope derived from the host cell membrane and thus become infectious mature virions [58].

## 3. Mechanisms by Which CHIKV Causes Chronic Arthritis

**Figure 4:**
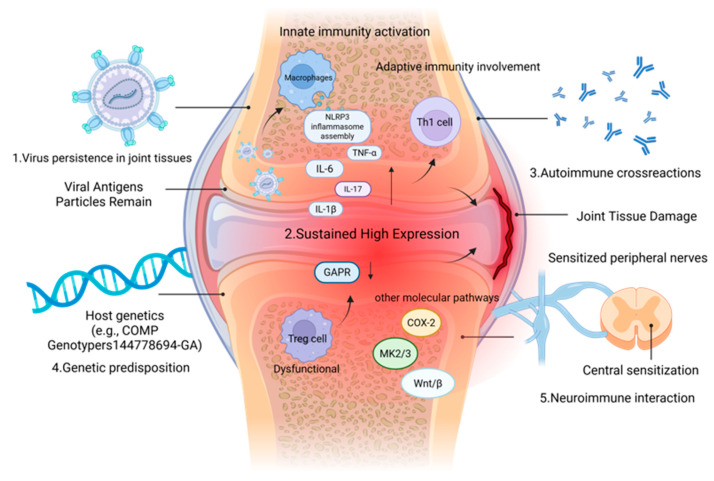
Pathogenesis of CHIKV-induced chronic arthritis. Persistent viral RNA in joint tissues triggers sustained activation of innate immunity, including NLRP3 inflammasome-mediated IL-1β and IL-18 release, and NF-κB-driven production of IL-6, TNF-α, and IL-17. This leads to Th1 and Th17 cell infiltration, Treg dysfunction, and the upregulation of COX-2, MK2/3, Wnt/β-catenin, and GAPR, resulting in synovial inflammation, cartilage degradation, and joint damage. Autoimmune mechanisms may also contribute to disease persistence. Created in BioRender. RUI, P. (2026) https://BioRender.com/s090pzx (accessed on 26 March 2026).

The pathogenesis of CHIKV-induced chronic arthritis, including the roles of innate and adaptive immunity, inflammatory cytokines, and key signaling pathways, is summarized in Figure 4. Arthritis after CHIKV infection presents a complex pathological process ranging from acute and severe onset to potentially prolonged and chronic. Usually within a week of being bitten by a virus-carrying Aedes mosquito, a sudden onset of high fever, often rising briefly above 39 °C, is accompanied by severe joint pain [59,60]. This pain often occurs symmetrically in the wrist, ankle, knee, and hand and foot small joints, most cases accompanied by obvious joint swelling, redness, and tenderness, forming a true arthritis [61,62]. Meanwhile, systemic symptoms such as severe myalgia, skin maculopapular rash, headache, and conjunctivitis are also common. Although systemic manifestations such as fever subside gradually within a week or two, joint discomfort may persist, often indicating a possible transition to a chronic phase of the disease.

In the chronic phase, the joint symptoms of many patients do not disappear with the end of acute viral infection, but last for months or even years, manifested as pain and inflammation in multiple joints, often accompanied by morning stiffness. The clinical characteristics of the disease are very similar to those of rheumatoid arthritis, and sometimes even meet the diagnostic criteria of rheumatoid arthritis, which brings difficulties to clinical differentiation [63]. This tendency to chronicity results from the complex interaction between the virus and the host immune system. At the molecular level, specific cytokines and signaling pathways dominate the process of chronic inflammation and joint destruction [64,65]. Lidbury, Brett A et al. proposed in 2008 that the main core mechanism of CHIKV-induced arthritis is that viral replication activates immune cells, releases pro-inflammatory factors, such as TNF-α and IL-1β, initiates local inflammatory response, and then activates NF-κB and other related inflammatory pathways to cause damage [66]. The critical nature of the innate immune response is emphasized. In addition to the inflammatory cytokine network, the interferon pathway plays a crucial role in the pathogenesis of Chikungunya virus infection. Type I interferons (IFN-α/β) are rapidly induced upon infection and are essential for controlling viral replication and dissemination. CHIKV has evolved mechanisms to evade the interferon response, mainly through the action of nsP2 and nsP3 proteins, which inhibit interferon signaling and the production of antiviral molecules. The impairment of interferon pathways is associated with increased viral persistence and the severity of chronic arthritis symptoms. Recent studies have highlighted that enhancing interferon responses may be a potential therapeutic strategy for CHIKV-induced chronic disease [67]. Subrat Thanapati, Shruti Kulkarni et al. (2025) also verified that chronic arthritis caused by CHIKV showed a persistently high expression of inflammatory mediators such as IL-1β, IL-6, GM-CSF and MCP-1, while IL-1β showed the most significant difference [68]. Meanwhile, Alessandro Conforti et al. pointed out that during the process of CHIKV infection evolving into chronic arthritis and the persistent presence of pro-inflammatory factors, IL-17 also exhibits differential expression [69]. Xiang Liu, Yee-Suan Poo et al. also confirmed that IL-17 is a key factor driving arthritis inflammation after CHIKV infection, and its deletion can significantly reduce joint pathological damage [70]. Camila Neiva Porto Silva et al. demonstrated that the release of proinflammatory cytokines such as IL-1β, IL-6, and TNF-α is due to the activation of the NLRP3 inflammasome by viruses, and proposed that chronic pain is not only caused by local inflammation in the joint, but also often involves a complex mechanism of neuroimmune interaction. The pain of many patients shows the characteristics of neuropathic pain such as burning sensation or pinprick sensation, and the response to conventional anti-inflammatory drugs is limited. This may be because long-term inflammatory mediators stimulate and sensitize peripheral nerves, and even cause nerve axonal damage. Continuous pain signals are transmitted to the central nervous system, which may also trigger central sensitization, thus maintaining the pain state for a long time [71,72]. This fills the gap between the CHIKV-induced inflammatory cascade and the central nervous system. In 2022, John E Dobbs et al. investigated 124 patients who had been infected with CHIKV four years earlier and collected blood samples to determine the total number of viable peripheral monocytes, CD4^+^ cells, T regulatory cells, and their immune markers. In a previous study, GARP protein on the surface of regulatory T cells was significantly decreased in CHIKV-induced chronic arthritis patients, and GARP plays a key role in the activation of anti-inflammatory factor TGF-β1. The expression of TGF-β1 is related to the function of the immune system [73]. In an earlier study by Yosra Bedoui et al., C-reactive protein and anti-CCP antibodies, which are major markers of inflammation and joint damage, were found to be upregulated in the serum of patients who developed chronic arthritis. Meanwhile, CHIKV induces chronic inflammation through the upregulation of COX-2, which is abundantly expressed in osteoclasts, indicating an imbalance between persistent immune activation and tissue repair [74]. Proteomic analysis by Althaf Mahin et al. showed that CHIKV would activate the MK2/3 signaling pathway, upregulate the expression of actin-binding proteins PFN1 and TAGLN2, and at the same time, cooperate with the reprogramming of purine metabolism, constituting the basic pathological mechanism of chronic arthritis [75]. More recently (2025), Mariana Severo Ramundo et al. used transcript sequencing analysis of blood samples obtained from patients during the acute and late phases of viral infection and hypothesized that patients who develop chronic arthritis have impaired interleukin signaling early in their illness. This was associated with the upregulated expression of has-miR-98-5p, and pCHIKV-CIJD patients showed reduced transcript levels of MMP8, LFT, and DDIT4 [76]. This provides a new target for the treatment of CHIKV-induced chronic arthritis. At the cellular level, Jian Li, Jiandong Guo et al. proposed that CHIKV can affect the differentiation function of mesenchymal cells and osteoblasts, and then affect multiple osteogenic signaling pathways such as the Wnt/β-catenin pathway, perturb the immune response, and participate in the process of joint tissue damage while clearing the virus. Beyond innate immunity, adaptive immune responses, particularly CD4^+^ T cells, play a pivotal role in the transition to chronic chikungunya. Infiltrating virus-specific CD4^+^ T cells have been identified as major pathogenic mediators that sustain joint inflammation. Furthermore, the mechanism of chronicity is closely linked to the “viral persistence” hypothesis. Viral RNA and antigens can persist in synovial macrophages and other joint-resident cells during the chronic phase, serving as a persistent trigger for local immune activation and inflammation [77]. In 2023, a study confirmed the relevance of the Wnt/β pathway [78,79]. Anthony Torres-Ruesta et al. proposed that virus-specific, infiltrating joint Th1 cells are the main pathogenic mediators of CHIKV-induced joint lesions [80]. At the genetic polymorphism level, Siddhartha Sengupta et al. genotyped 167 CHIKV patients and 102 healthy controls. Compared with the data, CRP, anti-CCP antibody, IL-2R and COMP levels were significantly increased in patients with CHIKV-induced chronic arthritis. AST, ALT, AST/ALT ratio, bilirubin, and ALP concentrations were also increased, and people with the COMP-rs144778694-GA genotype were more susceptible to infection [81]. Through omics analysis, Hongyu Chen, Kaiyun Ding et al. concluded that CHIKV infection-related arthritis was closely related to intestinal flora disorder and age-related differences in immune response [82]. This provides new ideas for diagnosis and treatment.

In summary, CHIKV-induced chronic arthritis is a disease that is initiated by direct viral damage, promoted by both innate and adaptive immunity, and may develop into a chronic condition due to the persistence of viral antigens, the initiation of autoimmune responses, and individual genetic susceptibility.

## 4. Prevention and Control of CHIKV

The major CHIKV vaccine candidates in clinical and preclinical development, along with their key characteristics and clinical trial outcomes, are summarized in Table 1. Currently, there are no specific antiviral drugs for the treatment of CHIKV, and only two vaccines have been approved by the U.S. FDA. VLA1553 was approved by the U.S. FDA in 2023. Based on a live attenuated vaccine platform, VLA1553 can trigger a strong immune response and has been confirmed to have good safety [83]. In addition, Vimkunya, a vaccine based on the VLP platform, was announced for marketing in 2025. In clinical trials, Vimkunya was demonstrated to elicit rapid and robust immune responses and was well-tolerated in adolescents [84]. Vimkunya has also been shown to induce protective serum levels with an acceptable safety profile in people over 65 years of age [85]. In addition to VLA1553, another vaccine based on the live attenuated vaccine platform, CHIKV/IRES, is currently in Phase I clinical trials and has been shown to provide good immune protection and a certain safety in AG129 mice in preclinical studies [86]. In addition to traditional vaccine platforms, a number of vaccines based on novel vaccine platforms have shown good performance in preclinical or clinical trials. ChAdOx1 Chik is a chimpanzee adenovirus vector vaccine based on the structural protein of CHIKV. In Phase I clinical trials, a single dose of chadOx1 CHIK vaccination elicited neutralizing antibodies against four lineages. Only mild injection site pain and headache were reported [87]. In addition to the chimpanzee adenovirus vector, another measles vector-based vaccine, MV-CHIK, also performed well in clinical trials. Neutralizing antibodies against chikungunya virus were detected in all MV-CHIK treatment groups after one or two immunizations. Geometric mean titers ranged from 12.87 (95% CI 8.75–18.93) to 174.80 (119.10–256.50), and no related serious adverse events were reported [88].

In recent years, novel mRNA vaccine platforms have been widely used due to their excellent performance during the COVID-19 pandemic. Mrna-1388 is a mRNA vaccine constructed by Moderna Company based on the full-length structural protein of the CHIKV West Africa 37997 strain. The neutralizing antibody titer was significantly increased after the second immunization, and the neutralizing antibody titer was still higher than that of the placebo group one year after the second immunization [89]. In addition to mrNA-1388, another mRNA vaccine based on the full-length structural protein of the CHIKV LR2006 OPY1 strain was demonstrated in preclinical studies to elicit strong pseudovirus neutralizing antibody titers in mice and elicit strong CD8+ cellular immune responses [90]. Notably, a recent mRNA vaccine based on the conserved full-length structural protein or a single E protein sequence of CHIKV has been shown to elicit strong humoral and cellular immune responses, and has been shown to provide good protection in multiple animal models [91].

## 5. Conclusions

Chikungunya virus has emerged as an important emerging infectious disease threat globally because of the increasing movement of people and the efficient transmission by mosquitoes and the potential public health burden of chronic arthritis that can result from infection. The extensive spread of the virus is the result of the adaptive evolution of its genome, the changes of vector ecology, and the globalization process. An in-depth understanding of the mechanisms of viral replication and pathogenesis, especially the immune and molecular basis of chronic arthritis (involving persistent inflammation, cytokine network disruption, neuroimmune interactions, and the critical role of interferon pathways), is the key to the development of effective intervention strategies.

Although treatment options are still limited, the rapid progress in vaccine research and development, especially the promising prospects of multiple technical platform vaccine candidates, brings hope to the prevention and control of CHIKV.

However, challenges remain, including the need for more effective antiviral drugs, improved surveillance, and a deeper understanding of the host–virus interaction, particularly the role of interferon signaling and genetic susceptibility in chronic disease progression. Future research should focus on these areas to develop comprehensive strategies for the prevention, treatment, and long-term management of chikungunya virus infection.

Chikungunya virus has emerged as an important emerging infectious disease threat globally because of the increasing movement of people and the efficient transmission by mosquitoes and the potential public health burden of chronic arthritis that can result from infection. The extensive spread of the virus is the result of the adaptive evolution of its genome, the changes of vector ecology, and the globalization process. An in-depth understanding of the mechanisms of viral replication and pathogenesis, especially the immune and molecular basis of chronic arthritis (involving persistent inflammation, cytokine network disruption, and neuroimmune interactions), is the key to the development of effective intervention strategies. Although treatment options are still limited, the rapid progress in vaccine research and development, especially the promising prospects of multiple technical platform vaccine candidates, brings hope to the prevention and control of CHIKV. In the future, it is necessary to continue to strengthen viral surveillance, in-depth study on the mechanism of chronic diseases, and promote the development and application of safe and effective vaccines and antiviral drugs to cope with this persistent health challenge.

## Figures and Tables

**Table 1:** Summary of the chikungunya virus (CHIKV) vaccine candidates in target and preclinical development.

Name (Candidate)	Platform	Target	Sponsor/ Agency	Trial ID (NCT)	Phase	Key Metrics (Efficacy/ Immunogenicity)
VLA1553	Live attenuated	/	Valneva Austria GmbH	NCT04546724	Marketed	98.9% seroprotection rate (SPR) at Day 28; sustained for 24 months
Vimkunya	VLP	Structural protein (C/E2/E1)	Bavarian Nordic	NCT05349617	Marketed	High nAb titers (GMT > 100) in 90%+ of participants across all age groups
CHIKV/IRES	Live attenuated	/	/	/	Phase I	100% protection in NHP models; well-tolerated with no viremia detected
ChAdOx1 Chik	Adenoviral vector	Full-length structural protein	University of Oxford	NCT03590392	Phase I	100% seroconversion after a single dose; robust T-cell response
MV-CHIK	Measles-vectored	Full-length structural protein	Themis Bioscience GmbH	NCT02861586	Phase II	GMTs: 12.87–174.80; 100% SPR after the second dose (booster)
mRNA-1388	mRNA	Full-length structural protein	ModernaTX, Inc	NCT03325075	Phase I	Significant nAb increase; persistence above placebo for 1 year post-dose 2
mCV1	mRNA	Full-length structural protein	/	/	Preclinical	Potent CD8+ T-cell response; 100% survival in lethal challenge models
mCV2	mRNA	Structural protein E	/	/	Preclinical	High E-protein specific IgG titers; reduced viral load in joint tissues.

## Data Availability

No new data were created or analyzed in this study. Data sharing is not applicable to this article.
